# THE BLACK BOX OF CARE MANAGEMENT IN MANAGED LONG-TERM SERVICES AND SUPPORTS

**DOI:** 10.1093/geroni/igac059.749

**Published:** 2022-12-20

**Authors:** Katherine Abbott, Jennifer Heston-Mullins, Athena Koumoutzis, Dayna Bennett, Karen Williams, Robert Applebaum

**Affiliations:** Miami University, Oxford, Ohio, United States; Scripps Gerontology Center, Miami University, Oxford, Ohio, United States; Miami University, Oxford, Ohio, United States; Miami University, Oxford, Ohio, United States; Miami University, Oxford, Ohio, United States; Miami University, Oxford, Ohio, United States

## Abstract

Within Ohio’s MyCare demonstration, two distinct care management models were selected by the participating MyCare Ohio health plans (MCOPs): fully-delegated waiver care management and waiver service coordination. The purpose of this presentation is to describe the components of care management operating in MyCare Ohio. Qualitative interviews with n=91 Area Agency on Aging (AAA) and n=131 MCOP care management personnel were audio-recorded, transcribed, and checked for accuracy prior to thematic coding in Dedoose. Results indicate that comprehensive care management is the core element of MyCare Ohio. Fully-delegated care management models were viewed by participants as beneficial to reducing confusion for members however ‘scope creep’ challenged the already strained AAAs. Effective teamwork was identified for waiver service coordination models but the division of labor and communication needed between the AAA and MCOP care management personnel created tensions. The discussion will focus on practice recommendations for training, caseloads, and support staff.

